# A structured telephone-delivered intervention to reduce problem alcohol use (Ready2Change): study protocol for a parallel group randomised controlled trial

**DOI:** 10.1186/s13063-019-3462-9

**Published:** 2019-08-19

**Authors:** Dan I. Lubman, Jasmin Grigg, Victoria Manning, Kate Hall, Isabelle Volpe, Stephanie Dias, Amanda Baker, Petra K. Staiger, John Reynolds, Anthony Harris, Jonathan Tyler, David Best

**Affiliations:** 1 Eastern Health Clinical School, Monash University, Box Hill, Australia; 2 Turning Point, Eastern Health, Richmond, Australia; 3 School of Psychology, Deakin University, Geelong, Australia; 4 Centre of Drug, Addictive and Anti-social Behaviour Research (CEDAAR), Deakin University, Melbourne, Australia; 5 School of Medicine and Public Health, University of Newcastle, Callaghan, Australia; 6 Faculty of Medicine, Nursing and Health Sciences, Monash University, Clayton, Australia; 7 Centre for Health Economics, Monash University, Clayton, Australia; 8 Department of Law and Criminology, Sheffield Hallam University, Sheffield, UK

**Keywords:** Alcohol, Substance use disorder, Randomised controlled trial, Telephone, Treatment, Intervention

## Abstract

**Background:**

Current population surveys suggest around 20% of Australians meet diagnostic criteria for an alcohol use disorder. However, only a minority seek professional help due to individual and structural barriers, such as low health literacy, stigma, geography, service operating hours and wait lists. Telephone-delivered interventions are readily accessible and ideally placed to overcome these barriers. We will conduct a randomised controlled trial (RCT) to examine the efficacy of a standalone, structured telephone-delivered intervention to reduce alcohol consumption, problem severity and related psychological distress among individuals with problem alcohol use.

**Methods/design:**

This is a single site, parallel group, two-arm superiority RCT. We will recruit 344 participants from across Australia with problem alcohol use. After completing a baseline assessment, participants will be randomly allocated to receive either the Ready2Change (R2C) intervention (*n* = 172, four to six sessions of structured telephone-delivered intervention, R2C self-help resource, guidelines for alcohol consumption and stress management pamphlets) or the control condition (*n* = 172, four phone check-ins < 5 min, guidelines for alcohol consumption and stress management pamphlets). Telephone follow-up assessments will occur at 4–6 weeks, 3 months, 6 months and 12 months post-baseline. The primary outcome is the Alcohol Use Disorders Identification Test (AUDIT) score administered at 3 months post-baseline. Secondary outcomes include change in AUDIT score (6 and 12 months post-baseline), change in number of past-month heavy drinking days, psychological distress, health and wellbeing, quality of life, client treatment evaluation and cost effectiveness.

**Discussion:**

This study will be one of the first RCTs conducted internationally to examine the impact of a standalone, structured telephone-delivered intervention to address problem alcohol use and associated psychological morbidity. The proposed intervention is expected to contribute to the health and wellbeing of individuals who are otherwise unlikely to seek treatment through mainstream service models, to reduce the burden on specialist services and primary care providers and to provide an accessible and proportionate response, with resulting cost savings for the health system and broader community.

**Trial registration:**

Australian New Zealand Clinical Trials Registry, ACTRN12618000828224. Pre-registered on 16 May 2018.

**Electronic supplementary material:**

The online version of this article (10.1186/s13063-019-3462-9) contains supplementary material, which is available to authorized users.

## Background

Alcohol consumption is the second leading cause of preventable morbidity and mortality in Australia, contributing to more than 100,000 hospitalisations and 3000 deaths each year [1] and increasing the risk for more than 60 different diseases and conditions [2]. Almost 50% of Australians are adversely affected by someone else’s drinking, costing those around them more than AUD$13 billion in out-of-pocket expenses and a reduced quality of life estimated at more than AUD$6 billion per annum [3]. Of the 20% of Australians meeting lifetime criteria for alcohol misuse (i.e. abuse or dependence), only one in five (22.4%) seeks help [4]. Co-occurring mental health issues, particularly anxiety and mood disorders, are also high [5]. While publicly funded treatment services are available in each state and territory, they have necessarily evolved to address the complex needs of the comparatively small minority of dependent drinkers falling at the severe end of the continuum (i.e. those with significant physical and mental health comorbidity and marked social disadvantage), often requiring expensive multi-disciplinary and inter-sectoral care. However, it is the substantially larger population of problem drinkers without complex medical or psychosocial needs, who are unlikely to seek treatment, who cause the greatest cost to society due to their sheer number [6]. As such, it is imperative that the available treatment options are broadened to address the entire continuum of problem alcohol use and that known barriers to treatment accessibility are addressed [7].

There is a substantial body of evidence on the effectiveness of brief interventions delivered in primary care settings for non-treatment-seeking people with problem alcohol use [8]. Structured, less intensive interventions can be used within a stepped care model, where individuals commence work on reducing their drinking before engaging in longer, more intensive programs or treatments, if needed [8]. There is also growing evidence for the benefit of multi-session brief interventions that typically address motivation, are solution focused and provide skills training, goal setting and craving management strategies [9–11]. Multi-component interventions which include integrated, evidence-based approaches (e.g. cognitive behavioural therapy and motivational interviewing) have been proposed to have an additive effect in treating problem alcohol use [12] as well as addressing comorbid mental health problems [9, 10, 13].

Most studies examining the effectiveness of brief interventions for problem alcohol use have been conducted in general health settings, particularly primary care, where problem alcohol use has been identified through opportunistic screening [14, 15]. Despite the effectiveness of such interventions, problem alcohol use remains poorly detected and treated within most healthcare settings [16–18]. Delivery of opportunistic interventions within primary care is sporadic at best, with multiple barriers to implementation in these settings (such as time, cost and lack of knowledge about when and how to implement such interventions) [19].

Additional barriers to accessing treatment for problem alcohol use include service accessibility (e.g. wait lists, service operating hours and difficulty in attending sessions scheduled at regular times in fixed locations) [20], geographical barriers (e.g. location, restricted transport options and scarcity of services particularly in regional/rural areas), the experience of self-stigma and concerns about anonymity. To overcome these barriers, attention has increasingly turned towards designing interventions that utilise different, flexible and more accessible modes of service delivery. Telephone-based support, such as 24/7 alcohol and drug helplines, are ideally placed to overcome many of the barriers to accessing treatment for problem alcohol use. Alcohol and drug helplines are available in every Australian state and territory, and they collectively respond to more than 140,000 calls each year [21]. The 24-h availability of such support offers increased accessibility for individuals in regional areas, as well as those requiring child care, those in full-time employment or those who feel stigmatised or have a preference for anonymity. These helplines are often the first point of contact for individuals seeking help for problem alcohol use. However, these inbound services traditionally provide a one-off response to the caller’s immediate request for assistance, with a focus on crisis support, information provision and referral to face-to-face treatment [21, 22].

This study will be one of the first to use randomised controlled methods to examine the efficacy of standalone, multi-session interventions for problem alcohol use delivered via telephone, despite evidence for the effectiveness of telephone-delivered interventions for other substances (e.g. tobacco and cannabis use) [23, 24]. A recent review of studies, of any methodological design, that examined telemedicine interventions for the treatment of substance use disorders concluded that this approach holds potential for effectiveness in reducing substance use [25]. Despite a paucity of research in the alcohol field, there is evidence from non-randomised research that proactive telephone-delivered interventions (i.e. outbound telephone counselling) are likely to be effective for problem alcohol use [23, 26]. With increasing support for the effectiveness of telephone-delivered interventions for a range of substance use problems, and growing concerns related to the increasing harms and costs associated with problem alcohol use across Australian communities, we will examine the impact of a multi-session, structured telephone-delivered intervention (Ready2Change, R2C [27]) to address problem alcohol use (and associated psychological morbidity) in a randomised controlled trial (RCT).

### Choice of comparator

Evidence from primary care settings (and some evidence from other healthcare settings) suggests that screening and a single occurrence of brief health education advice — either verbally or through the provision of self-help materials — yields short-term improvements in individuals’ alcohol consumption [15, 28, 29]. Data from several studies suggest that mere exposure to baseline questions about alcohol use can impact positively on alcohol consumption levels [30]. Baseline questioning effects may operate through similar mechanisms, prompting reflection and the self-regulation of behaviour [31]. Still, pooling effect sizes over large numbers of alcohol BI studies have been able to provide a powerful estimate of how this form of health promotion performs [15, 30, 32].

Therefore, to control for the effects of alcohol use assessment and frequency of contact, participants in the control group will receive brief check-in calls and be provided with health information pamphlets (see Additional file 2: Alcohol consumption pamphlet and Additional file 3: Stress management pamphlet). While participants in the control condition may experience some benefit from participation, we expect it will be less than that obtained for the active condition.

### Objectives

**Fig. 1:**
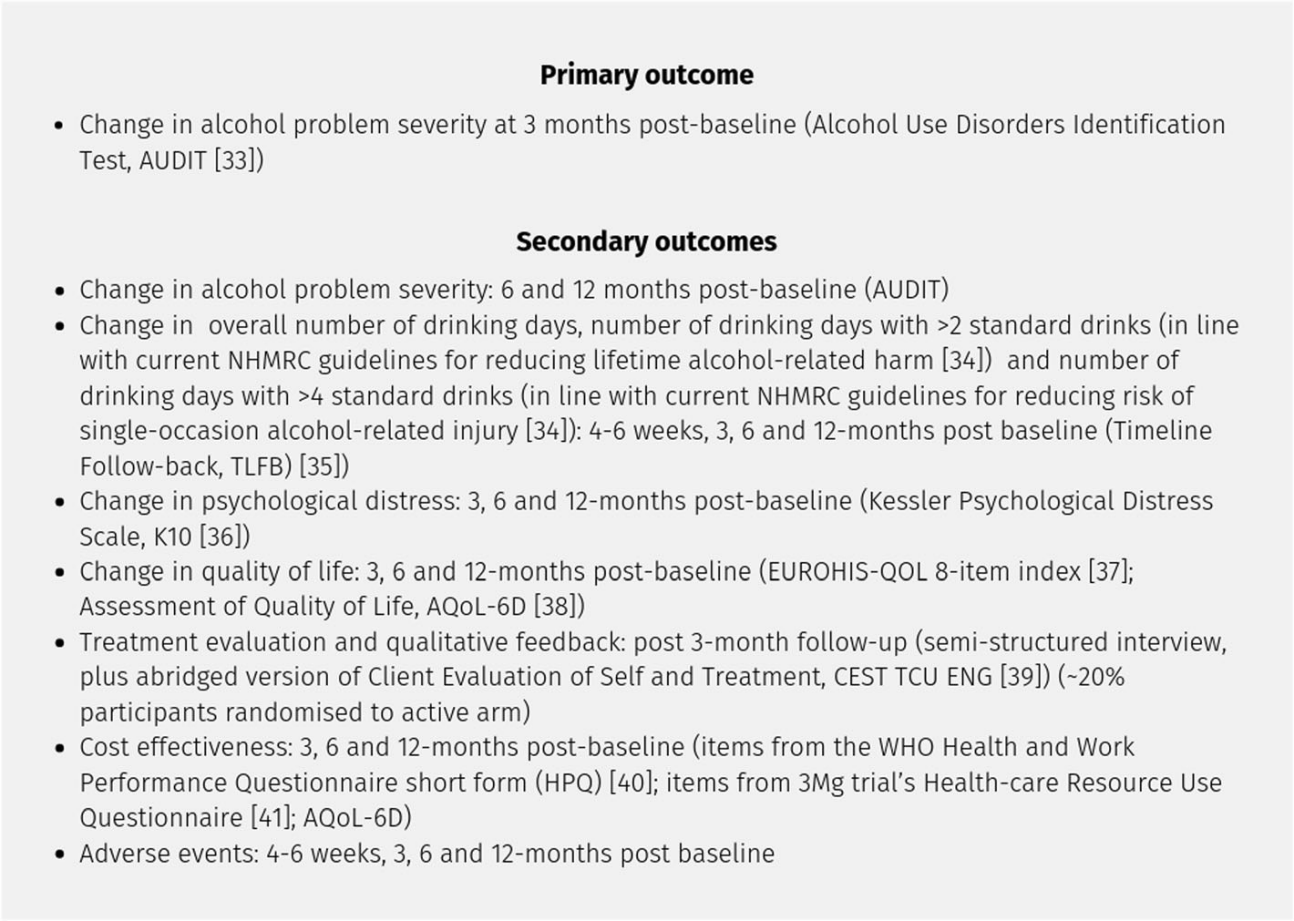
Primary and secondary outcomes [34–41]

The aim of the study is to examine the efficacy of the R2C telephone-delivered structured intervention in reducing alcohol problem severity and related psychological distress in individuals with problem alcohol use (defined here as an Alcohol Use Disorders Identification Test (AUDIT) score of > 6 for females and > 7 for males) [33], compared with the provision of basic health information and weekly check-in calls (see Fig. 1).

### Trial outcomes

Primary and secondary outcomes for this trial are detailed in Fig. 1.

## Methods

### Design

**Fig. 2:**
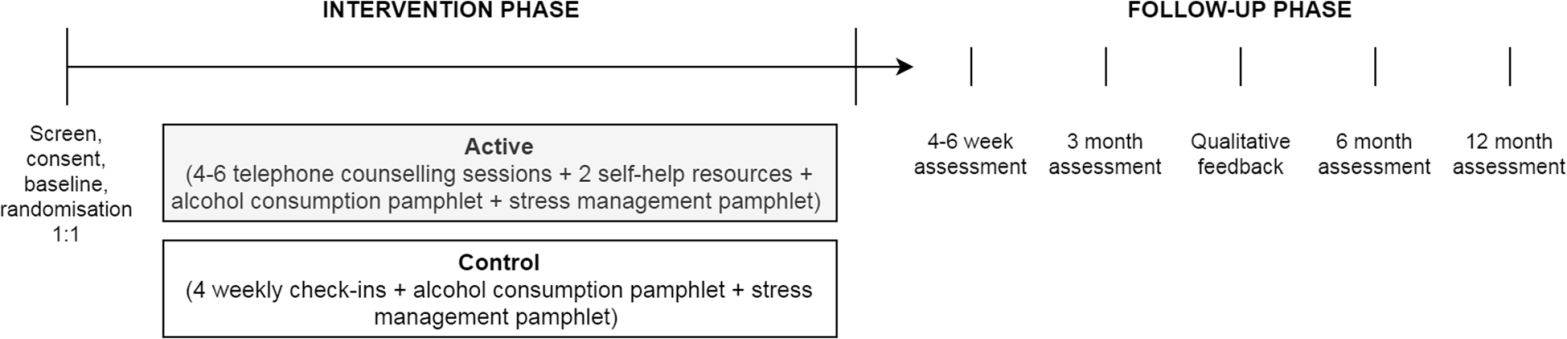
Study design

This study is a single site, parallel group, two-arm superiority RCT, with participants randomly allocated to receive either the R2C intervention or the control condition (Fig. 2). The protocol follows Standard Protocol Items: Recommendations for Interventional Trials (SPIRIT) guidelines (see Table 1: SPIRIT figure and Additional file 1: SPIRIT checklist).

**Table 1:** SPIRIT table

			Intervention		Follow-up				
	Screening	Baseline	R2C	Control	4-6 week	3 month	Feed-back	6 month	12 month
Contacted by	R1	R1	Counsellor	R1	R2	R2	R1	R2	R2
Week	0	0	1-6	1-4	4-6	-	~13	-	-
Eligibility screen	X								
AUDIT	X					X		X	X
SADQ-C	X								
SIDAS	X								
Informed consent	X								
Allocation		X							
TLFB		X			X	X		X	X
Substance use		X				X		X	X
K10		X				X		X	X
EUROHIS-QOL 8-item index		X				X		X	X
Adverse events		X	X	X	X	X	X	X	X
Barriers to help-seeking		X							
Additional treatment enquiry					X	X		X	X
AQoL-6D		X				X		X	X
WHO HPQ		X				X		X	X
Cost data		X				X		X	X
Counselling sessions			X						
Self-help resources			X						
Information pamphlets			X	X					
CEST							X		
Qualitative feedback							X		

*R1* Researcher 1, *R2* Researcher 2

### Setting

The study is being conducted at Turning Point, a national addiction treatment and research centre in Melbourne, Victoria. Turning Point provides a range of clinical specialist treatment services for people affected by alcohol and other drugs across Australia, including a 24-h telephone counselling, information and referral service.

### Participants

A total of 344 participants (172 participants per trial arm) will be randomly allocated to one of the two study conditions.

### Inclusion/exclusion criteria

**Fig. 3:**
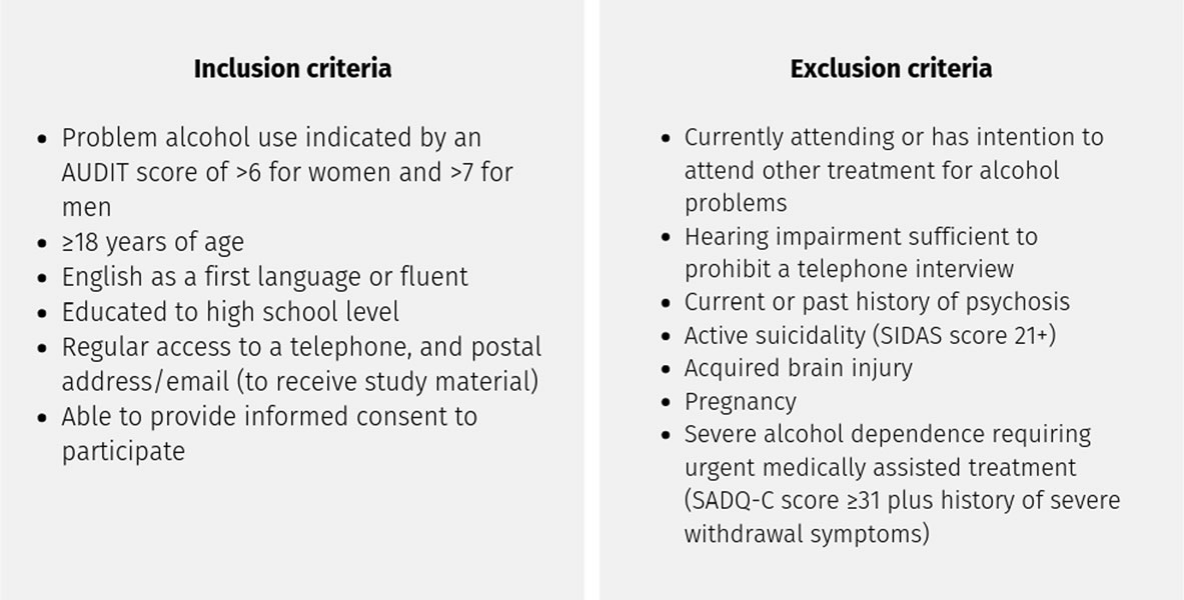
Participation criteria

The inclusion and exclusion criteria are described in Fig. 3.

### Recruitment and screening

All assessments will be conducted over the telephone, using protocols developed in our pilot studies and previous cohort work [42]. A research assistant (Researcher 1) will perform eligibility assessments and baseline data collection (see Table 2). A second research assistant (Researcher 2) will perform follow-up data collection. Participants, Researcher 2 and the study statistician will remain blind to participant allocation.

**Table 2:** Trial measures

	Data collected	Method
Screening measures	Demographic information *Structured questions*	Standard demographic characteristics (e.g. age, gender, education level)
	Problem alcohol use *AUDIT*	Assessed by the Alcohol Use Disorders Identification Test (AUDIT) [33]. A score of > 6 for women and > 7 for men warrants inclusion in the study
	Severity of alcohol dependence *SADQ-C*	Assessed by the Severity of Alcohol Dependence Questionnaire (SADQ-C) [54]. A score of 31 or higher indicates “severe alcohol dependence” and warrants exclusion from the study
	Presence/severity of suicidal thoughts *SIDAS*	Assessed by the Suicidal Ideation Attributes Scale (SIDAS) [55]. A score of 21+ indicates high risk of suicidal behaviour and warrants exclusion from the study
	Other inclusion/exclusion *Structured questions*	Structured questions querying self-reported information pertaining to the study’s inclusion/exclusion criteria (e.g. history of psychosis)
Outcome measures		
Primary outcome	Alcohol problem severity *AUDIT*	The primary outcome is alcohol problem severity at 3 months, assessed by the AUDIT. The time frame has been adapted to cover month prior to assessment (rather than year), so that planned follow-up assessments at 3, 6 and 12 months can be performed. Five short items from the recent Australian Institute of Health and Welfare (AIHW) National Drug Strategy Household Survey have been included to provide additional information on self-reported change in alcohol use and alcohol literacy
Secondary outcomes	Alcohol use patterns *TLFB*	Past-month (30 days) alcohol consumption and heavy drinking days, assessed by the Alcohol Timeline Follow-back (TLFB) [35]. Heavy drinking days are measured as > 40/60 g of alcohol (4/6 standard drinks)
	Substance use *AOD Self-Completion Form*	Assessed using items from the Victorian Department of Health and Human Services (DHHS) Victorian AOD Self-Completion Form [56], recording recent use of other drugs and frequency of use in the past 28 days
	Psychological distress *K10*	Assessed by the Kessler Psychological Distress Scale (K10) [36]
	Quality of life *EUROHIS-QOL 8-item index* *AQoL-6D*	Assessed by the EUROHIS-QOL 8-item index [37, 57] and Assessment of Quality of Life (AQoL-6D) [38]
	Cost effectiveness *AQoL-6D* *WHO HPQ* *Health-care* *Resource Use* *Questionnaire*	The incremental cost of treatment will be compared to the incremental benefits of treatment in terms of the primary outcomes and the difference in quality-adjusted life years (QALYs; assessed by the AQoL-6D). Work performance and productivity will be assessed by items from the WHO Health and Work Performance Questionnaire short form (WHO HPQ) [40]. Healthcare resource use is assessed using items from the 3Mg trial’s Health-care Resource Use Questionnaire [41]
	Adverse events *Structured questions*	During baseline, scheduled intervention/control calls, and follow-up calls, participants will be asked general questions: *“Have you had any health concerns recently* (baseline)/*since the last time you had telephone contact for this study?”*, *“Have you felt unwell or different since the last time you had telephone contact for this study?”* and *“Do you have any specific worries or complaints about your health in general?”*, as well as more specific questions: *“Has your alcohol use changed?”* and *“Has your mood changed?”*
Additional measures/participant feedback	Barriers to help-seeking *Structured questions*	Perceived barriers to treatment-seeking for alcohol problems will be identified through an open-ended question (*“What are the reasons that you have not sought treatment for your alcohol use in the past?”*) and from a list of 15 barriers (e.g. financial, stigma, readiness for change) developed by Schuler et al. [58]
	Treatment participation and satisfaction *CEST TCU ENG*	Assessed by the Client Evaluation of Self and Treatment (CEST) short form (TCU ENG) [39] (approximately 20% of the R2C intervention group)
	Qualitative feedback *Structured questions*	Questions asked will include: *“Which parts, if any, of the telephone intervention sessions were most helpful to you? How were these most helpful?”* and *“What did you think about the delivery, format and duration of the telephone intervention sessions?”* (approximately 20% of the R2C intervention group)
	Additional treatment enquiry *Structured questions*	Engagement in any additional treatment (e.g. inpatient/outpatient treatment) or mutual aid group attendance (AA, SMART Recovery) will be queried and recorded for all participants (exclusion criterion at screening only)

To recruit our target sample size, recruitment methods will be broad. Participants will be recruited from across Australia via a number of channels, including print, radio and online advertising (including social media), clinician referrals, helpline service referrals (i.e. interstate Alcohol and Drug Information Services that do not currently offer R2C-type outbound interventions) and opportunistic study promotion. Online advertising will be the primary recruitment strategy due to its broad reach and cost effectiveness [43]. In the case of online advertising, individuals will be linked to a secure Qualtrics form in which to enter their contact details. Individuals who express interest in participating will be called by Researcher 1 and verbally provided with detailed information about the study before being assessed for eligibility to participate. Those who are eligible will be provided with the Participant Information and Consent Form (PICF) and asked to provide verbal consent to participate by Researcher 1. Baseline data collection can be undertaken during this phone call or scheduled for a different time as preferred by the participant. Participants will be randomised on completion of the baseline assessment.

### Randomisation

Participants will be randomly assigned to the R2C group or the control group with a 1:1 allocation ratio. Randomisation will be stratified by gender and will use a standard computer-generated “permuted blocks of variable size” scheme for each stratum. Randomisation lists for each stratum will be generated at the start of the study by the study statistician and linked to a unique identification code. The statistician who prepares the lists will play no other role in the delivery of the interventions. Allocations will be concealed in individual envelopes labelled with the unique identification code and opened (in consecutive order) by the designated researcher (Researcher 1) after the baseline assessment. Envelopes were used because at the time of trial design and start-up it was not feasible to implement a centrally managed, online randomisation system. The contents of an envelope cannot be discerned without opening the envelope, and monitoring of randomisation dates and within-stratum sequence numbers is conducted to ensure randomisation errors do not occur. Randomised participants will be assigned their unique identification code, which will be in a re-identifiable format. Participants’ identifying information collected during this study will be stored separately from trial data, while the unique identifying code will be attached to both. Participant information and trial data will be stored in separate locked filing cabinets located on the study premises.

Following randomisation, participants will be contacted by Researcher 1 and provided with an overview of their assigned protocol (i.e. R2C or control). Participants will not be told any details about the other intervention. Appropriate resources will be sent to the participant (*R2C intervention*: R2C self-help booklets [44, 45] + pamphlets [34, 46]; *control*: pamphlets [34, 46]). Hard copy materials will be posted to the participant (soft copy materials will be sent via email at the participants’ preference). An outbound telephone call will be scheduled within 7 days to commence the four to six R2C sessions with the participant’s dedicated R2C Counsellor (*intervention*), or to commence the four phone check-ins with Researcher 1 (*control*). The treatment period starts within 7 days of randomisation.

### Intervention

**Fig. 4:**
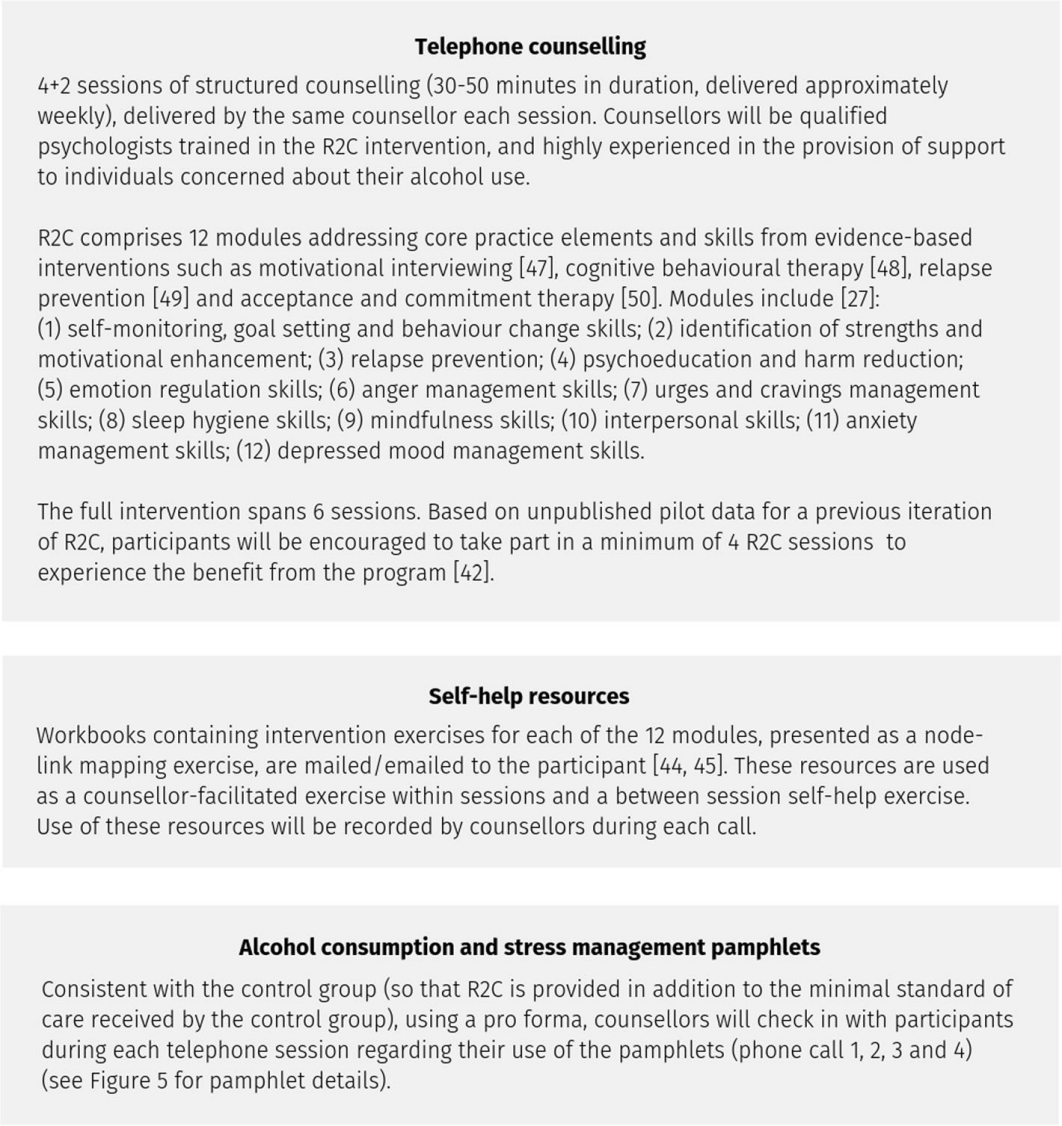
R2C intervention [27, 42, 44, 45, 47–50]

Participants randomised to the R2C intervention will receive telephone counselling (incorporating evidence-based interventions [47–50]), self-help resources and pamphlets (Fig. 4).

**Fig. 5:**
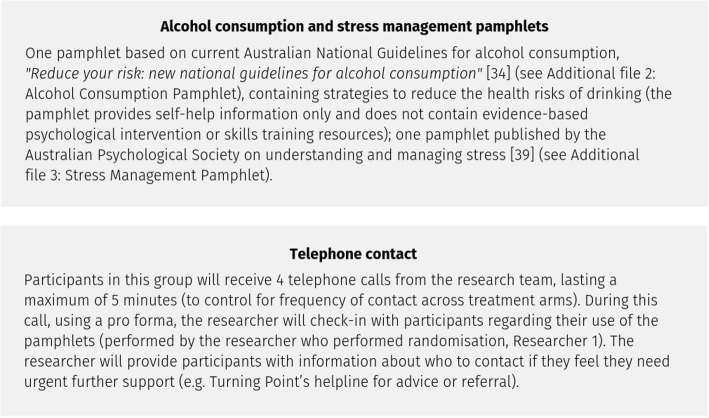
Control condition

Participants randomised to the control conditions will receive pamphlets and telephone contacts (Fig. 5).

In both the treatment and control conditions, the call duration will be recorded. Short Message Service (SMS) messages will be sent to remind participants of all pre-arranged telephone sessions, and five attempts at contact within 1 week will be made per scheduled phone session. R2C intervention calls will be made from Turning Point’s telephone and online services department, which is equipped with the infrastructure needed for outbound calls and call recording.

### Follow-up

Follow-up assessments will be conducted by Researcher 2. The first follow-up assessment will be performed at 4–6 weeks post-baseline. Follow-up assessments will also be performed at 3, 6 and 12 months. The assessment at 4–6 weeks post-baseline will take approximately 10 min to complete (Timeline Follow-back [TLFB], adverse events and additional treatment enquiry only). The next three follow-up assessments will take approximately 30 min to complete. For each follow-up, five attempts to contact the participant will be made within 2 weeks. Approximately 20% of the intervention group, selected using random start systematic sampling, will be asked to complete additional qualitative feedback post the 3 months follow-up.

### Loss to follow-up

Participants who cannot be contacted after five phone calls will be deemed to be missing at that data collection time point. Researcher 2 will attempt to contact participants again at the next data collection time point, following the same procedure, unless the participant actively withdraws.

### Retention

The study will use retention enhancement techniques suggested by previous studies [51, 52] to encourage retention in the intervention calls and follow-up assessments. Retention enhancement techniques will include active verbal commitment, flexibility in scheduled call times, weighting reimbursements according to the importance of collecting data at each time point and text message reminders before scheduled calls.

### Reimbursement

Participants will be reimbursed with vouchers as follows: AUD$20 for baseline assessment, AUD$20 for 4–6 weeks assessment, AUD$40 for 3 months assessment (primary outcome time point), AUD$20 for 6 months assessment, AUD$40 for 12 months assessment (last assessment) and AUD$10 for treatment evaluation/qualitative feedback (for those who are selected).

### Data collection

An electronic Case Report Form (eCRF) will be completed for each participant using REDCap (Research Electronic Data Capture [53]), summarising all screening and study data. REDCap is a secure, web-based application designed to support data capture for research studies (including case report forms, real-time data entry validation and audit trails).

### Trial monitoring

#### Treatment integrity

Training and supervision to ensure treatment fidelity will include a week-long training session focusing on competence and adherence to the R2C intervention and research procedures, as well as regular clinical supervision. All sessions will be digitally recorded, and an independent research assistant will rate fidelity of intervention sessions for 20% of participants using random start systematic sampling. During each intervention session, R2C Counsellors will complete a checklist of modules, exercises and homework activities completed, to assist with adherence with the intervention.

Training will be provided to Researcher 1 (who completes the control group telephone check-ins), and a script will be used to ensure that only past-week use of pamphlets is queried (i.e. no inadvertent individualised counselling). Supervision of Researcher 1 will occur to prevent “drift”, and call duration records will be checked intermittently.

#### Adverse events

All adverse events will be recorded between the time of consent and the final follow-up visit. Participants will be asked about adverse events at baseline, during each R2C session/control check-in and at each point of follow-up contact. Participants will also be encouraged to contact the research team if they are concerned about an adverse event.

#### Participant assessed as at risk of suicide

When a participant is assessed as being at high risk of suicidal behaviour (a Suicidal Ideation Attributes Scale [SIDAS] score of 21+), referral to appropriate support is immediate. Researchers are trained in the National Centre for Suicide Prevention Training two-day Applied Suicide Intervention Skills Training (ASIST) course to ensure they are equipped with the skills to respond to suicide risk. Researchers are trained to transfer the participant to an on-duty Turning Point helpline counsellor when immediate risk is identified in order for risk to be managed and responded to in accordance with the clinical service’s clinical protocol. R2C Counsellors are well trained and experienced in the management of suicide risk, and clinical governance of risk for the trial is managed in accordance with organisational risk management procedures.

#### Participant withdrawal/discontinuation

The right to withdraw without consequence is outlined during the consent process and in the PICF. Verbal revocation of consent can be provided by the participant, or a Revocation of Consent form can be completed, with the option to remove all of the participant’s previously collected data or just to remove consent for further data collection. No further contact with the participant will be initiated by the research team upon verbal or written revocation of consent.

In instances where it has been identified that a participant meets exclusion criteria during the study (e.g. active suicidality), and/or that it is not in the best interests of the participant to remain in the study, the Principal Investigator (a psychiatrist) will decide whether to withdraw the participant from the trial. If a participant is withdrawn by the Principal Investigator, the reasoning for this will be explained to the participant. The participant will be referred to the appropriate clinical services for treatment and support. No further data collection will occur, with the exception of the details regarding adverse events.

### Statistical methods

#### Sample size estimation

A pilot study of an earlier version of the telephone-delivered intervention examined outcomes to a 3 month follow-up [42]. Thirty-four individuals concerned about their drinking were referred to the program and completed an average of 5.5 sessions. Participation in the intervention demonstrated significant reduction in alcohol problem severity (AUDIT score) and improvement in psychological distress (Kessler Psychological Distress Scale [K10] score). Treatment satisfaction and counsellor rapport scores were comparable to those found in face-to-face treatment. Using data from the pilot work, we found that the between-subject variance component in the AUDIT score was 23.8, the within-subject variance component was 49.8 (intraclass correlation [ICC] = 0.323) and the estimated improvement (decline) in the AUDIT score was 11.2 (standard error [SE] = 1.69). The calculation of the sample size used these estimates of the variance components, from the single-arm pilot study, and was based on the power of the *F* test for the overall time by treatment arm interaction and the power of the *t* test for the interaction contrast of primary interest, namely, baseline versus 3 months by treatment arm; calculations used the “apower” procedure for the non-central *F* distribution, and the “cut” function for the non-central *t* distribution in the GenStat statistical package [59].

We estimate, conservatively, that by 3 months, there will be an improvement (reduction of at least 8 on the AUDIT score) in the R2C arm and that the control arm could exhibit a modest improvement of 4. If these improvements are sustained at 6 and 12 months, then with 120 evaluable subjects in each arm and assuming independence between subjects and equicorrelation within subjects, the *F* test, conducted at the 5% significance level, for this treatment-by-time interaction will have 90% power (and the two-sided, 5% level, *t* test for the interaction contrast at 3 months will have 85% power). If these conjectured improvements by 3 months are not durable and, for example, deteriorate by 50% at 6 months, and the scores return, on average, to their baseline levels at 12 months, then this treatment-by-time interaction scenario will be detected with 85% power, and the power of the two-sided, 5% level *t* test for the interaction contrast at 3 months (the analysis of the primary outcome) remains unchanged at 85%.

The target sample size has been inflated from 120 per arm to 172 per arm to allow for approximately 30% drop-out, which is based on the attrition rate reported in a Swedish helpline study [26] and the experiences of the Chief Investigators where attrition in trials of face-to-face psychosocial interventions with this population average around 20% at 12 months [13].

#### Statistical analysis plan

Data will be collated, cleaned and validated, using programmed edit checks, in a database that will be locked prior to the unblinding of the study statistician. The primary analysis will take place after all subjects, not known to have withdrawn or not deemed lost to follow-up, have had their 12 months assessments, based on the intention-to-treat principle (i.e. subjects’ data are analysed as randomised and as stratified). A “per-protocol” sensitivity analysis will be restricted to those subjects with at least one post-baseline assessment and, for subjects randomised to the R2C arm, participation in at least one structured telephone counselling session.

The repeated measurements of the outcome variables will be analysed by fitting linear mixed models, with fixed effects for treatment and time, and their interaction, and random effects for subjects and assessments within subjects, using restricted maximum likelihood (REML). As well as accommodating missing values under the missing at random assumption, this method will allow the most suitable variance-covariance model for the repeated measures to be selected, using Akaike’s information criterion [63], and commonality of nonlinear trends over time to be explored via splines. The *F* test will be used to test for an overall group-by-time interaction, and the primary comparison, between groups, of their changes from baseline to 3 months follow-up will be based on a *t* test of the corresponding interaction contrast — this *t* test will utilise the predicted means and their variance-covariance matrix that are recovered from the fitted mixed model. Diagnostic plots of residuals will be assessed and, if deemed necessary, variance-stabilising transformations, such as the empirical logistic transformation, will be applied to the outcome variables and inferences will be based on the analyses conducted on the transformed scale. In a series of exploratory analyses, mixed models with covariates for gender, illicit drug use, extent of exposure to the intervention, exposure to other treatments or programs, level of psychological distress and, as appropriate, level of alcohol use at baseline will be fitted, including their interactions with treatment group, in order to identify moderating factors. A sensitivity analysis, to assess the impact of missing 3-month data on the primary outcome, will use a “tipping point” approach [63]. Categorical, ordinal and binary outcomes will be analysed in a similar way using generalised linear mixed models (GLMMs). The complete list of candidate covariates and details of the analyses will be specified in a statistical analysis plan that will be reviewed and approved by the Principal Investigator prior to database lock. Analyses will be conducted using the most appropriate procedures in GenStat, R and STATA.

#### Cost effectiveness

Economic evaluation will assess the incremental cost of R2C compared to the control. The incremental cost will be compared to the incremental benefits of treatment in terms of the primary outcomes and the difference in quality-adjusted life years (QALYs). The incremental QALYs will be measured by the between-group difference in the mean Assessment of Quality of Life – 6D (AQoL-6D) score over 12 months. A social perspective on costs will be taken and will include resource use incurred in the delivery of the helplines as well as health services irrespective of payment source. Healthcare costs will be calculated from the utilisation data and average unit costs for each item. Running but not training costs will be included in the primary analysis. The inclusion of time/productivity gains is controversial, and the cost-effectiveness ratios will be calculated with and without these “indirect costs” in the primary analysis, but a secondary analysis will include the money value of time lost from work and from lower productivity while at work using the World Health Organization (WHO) Health and Work Performance Questionnaire (HPQ). Confidence intervals for incremental cost effectiveness will be calculated directly using non-parametric bootstrapping. In addition, we will calculate a cost-effectiveness acceptability curve for a range of hypothetical money values of outcomes, based on individual cost and outcome differences between groups over the 12 months, using mixed linear regression modelling adjusted for baseline values of outcome and gender.

#### Dissemination and translation plan

The results of this trial will be disseminated to academic and health professional audiences via peer-reviewed publications and conference presentations. Participants will be informed they can access the Turning Point website for a summary report of trial findings in 2020. We will provide the results to the public via a press release and relevant sector newsletters. The outcomes of this research are expected to inform policy development by providing evidence of a practical, low-cost population-level approach to reduce problem alcohol use. The trial results will also be communicated to policymakers with the aim of implementing the program nationally (R2C is currently available in Victoria, Australia).

## Discussion

The outcomes of this project are expected to make a significant contribution to the health and wellbeing of Australians who are otherwise unlikely to seek treatment from specialist services, as well as generate substantial cost savings for the health system and broader community. Given that the proposed telephone-delivered model has already been piloted within an existing helpline service, we expect the feasibility of the proposed study to be high. Co-location between the research team and an existing national treatment service is a major strength of this study. This allows for close monitoring of study protocol adherence and for immediate feedback between the research team and counsellors. With a research-to-practice gap evident in translational health research, and a significant lag time between implementation of treatments shown to be effective in research (up to 15 years), this co-location also provides a unique opportunity to allow the findings to be quickly disseminated and implemented.

The study addresses the ethical issue of clinical equipoise. There is evidence to suggest that “ultra-brief” single-session interventions are effective in reducing problem alcohol use [60], which may be as little as the provision of information pamphlets [61], monitoring [62] or asking about one’s alcohol use [31]. There is currently uncertainty around the relative benefit of the extended R2C intervention, although it is expected that this multi-session model will result in improved outcomes. Additionally, although current engagement in treatment/intent to seek treatment is an exclusion criterion for the study, participants are not prohibited from seeking treatment after enrolment. Given that participants will receive detailed assessment, information pamphlets, monitoring for adverse events throughout the study and provision of referral information where necessary (i.e. appropriate helplines or advice to speak with a general practitioner), it is possible that participants randomised to the control group will still receive a standard of care that may assist them to reduce their alcohol consumption.

This will be one of the first RCTs internationally to examine the efficacy of a standalone telephone-delivered intervention for problem alcohol use. The outcomes of this study are likely to inform the delivery of interventions for a range of other health conditions, particularly those where help-seeking is low, stigma is high or early intervention is a priority (e.g. illicit drug use, gambling, mental health problems). The proposed model being tested has the potential to reduce the burden on specialist addiction treatment services and provide a more appropriate and proportionate response to problem alcohol use. The model also provides potential for significant cost savings by intervening before progression to a greater severity or chronicity of problem alcohol use.

### Trial status

This trial is at protocol version 4, dated 27 February 2018. Recruitment commenced on 25 May 2018. To date, 285 participants have been randomised. Recruitment of participants is expected to be completed by November 2019 (with the last 12 months follow-up to be completed in November 2020).

## Additional files

Additional file 1: SPIRIT 2013 checklist: recommended items to address in a clinical trial protocol and related documents. (DOC 121 kb)

Additional file 2: Alcohol Consumption Pamphlet. (PDF 245 kb)

Additional file 3: Stress Management Pamphlet. (PDF 113 kb)

## Data Availability

Not applicable.

## Abbreviations

AQoL-6D: Assessment of Quality of Life - 6D (scale)
AUDIT: Alcohol Use Disorders Identification Test
CEST: Client Evaluation of Self and Treatment
eCRF: Electronic Case Report Form
K10: Kessler Psychological Distress Scale
QALY: Quality-adjusted life year
R2C: Ready2Change
RCT: Randomised controlled trial
REDCap: Research Electronic Data Capture
SADQ-C: Severity of Alcohol Dependence Questionnaire
SIDAS: Suicidal Ideation Attributes Scale
TLFB: Timeline Follow-back
