# Supplementary material for: A structured telephone-delivered intervention to reduce problem alcohol use (Ready2Change): study protocol for a parallel group randomised controlled trial
Source: Trials. 2019 Aug 19;20:515. doi: 10.1186/s13063-019-3462-9 (PMC6701125; doi:10.1186/s13063-019-3462-9)

IF YOU OR SOMEONE YOU KNOW NEEDS SUPPORT AND TREATMENT TO REDUCE YOUR ALCOHOL INTAKE, YOU SHOULD CONTACT:

- Your doctor
- Your local community health service
- An alcohol or other drug helpline in your State/Territory:

ACT	(02) 6205 4545
NSW	(02) 9361 8000 (Sydney)<br>1800 422 599 (NSW country)
NT	(08) 8922 8399 (Darwin)<br>(08) 8951 7580 (Central Australia)<br>1800 131 350 (Territory wide)
QLD	1800 177 833
SA	1300 131 340
TAS	1800 811 994
VIC	1800 888 236
WA	(08) 9442 5000 (Perth)<br>1800 198 024 (WA country)

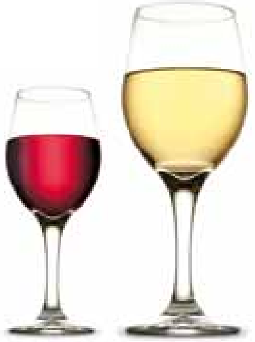

The advice in this brochure does not replace advice from your health care provider.

For more information about the new Australian Guidelines to Reduce Health Risks from Drinking Alcohol go to www.alcohol.gov.au

Australian Government
Department of Health and Ageing

STANDARD DRINK GUIDE

BEER

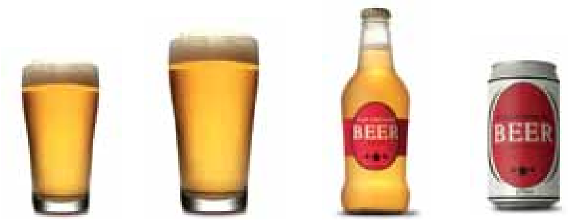

1.1 285ml Full Strength 4.8% Alc. Vol

1.6 425ml Full Strength 4.8% Alc. Vol

1.4 375ml Full Strength 4.8% Alc. Vol

1.4 375ml Full Strength 4.8% Alc. Vol

WINE

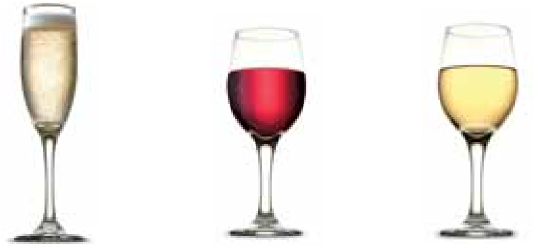

1.4 150ml Average Restaurant Serve of Sparkling Wine 12% Alc. Vol

1.6 150ml Average Restaurant Serving of Red Wine 13.5% Alc. Vol

1.4 150ml Average Restaurant Serving of White Wine 11.5% Alc. Vol

SPIRITS

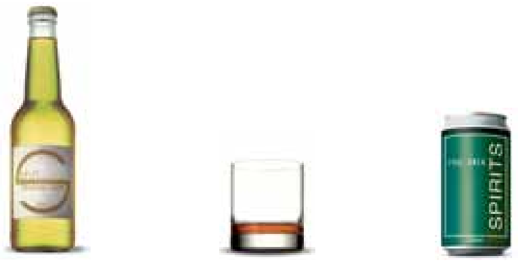

1.2 330ml Full Strength Ready-to-Drink 5% Alc. Vol

1 30ml High Strength Spirit Nip 40% Alc. Vol

1.5 375ml Full Strength Pre-mix Spirits 5% Alc. Vol

10/09

REDUCE YOUR RISK

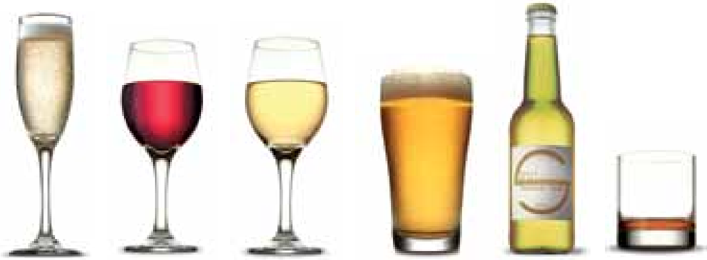

NEW NATIONAL GUIDELINES FOR **ALCOHOL CONSUMPTION**

New national guidelines for alcohol consumption have been developed by the National Health and Medical Research Council to help you reduce your risk of harm from alcohol.

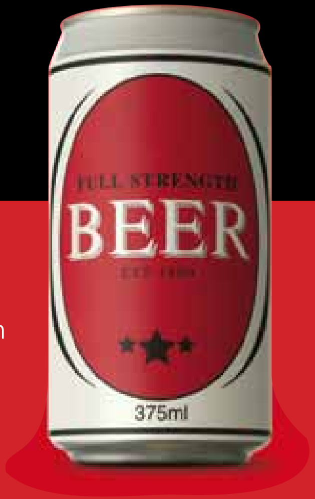

The guidelines are based on the most current and best available scientific research and evidence.

How much you drink is your choice, but the guidelines can help you make informed choices and help keep your risk of alcohol-related accidents, injuries, diseases and death, low – both in the short and long term.

WHAT DO I NEED TO KNOW?

Factors such as gender, age, mental health, drug use, and existing medical conditions can change how alcohol affects you. Responsible drinking is about balancing your enjoyment of alcohol with the potential risks and harm that may arise from drinking – especially if you go beyond low risk drinking levels.

WHAT DO THE GUIDELINES RECOMMEND?

- For healthy men and women, drinking **no more than two standard drinks on any day** reduces your risk of harm from alcohol-related disease or injury over a lifetime.
- Drinking **no more than four standard drinks on a single occasion** reduces the risk of alcohol-related injury arising from that occasion.

WHAT ARE THE HEALTH RISKS?

The health risks that accumulate over a lifetime from alcohol increase progressively – this means that the more you drink, the greater the risk.

Drinking alcohol can affect your liver or cause brain damage, heart disease, high blood pressure and increases your risk of many cancers. It may also increase your risk of injury through road trauma, violence, falls and accidental death.

WHAT IS A STANDARD DRINK?

A standard drink contains 10 grams of pure alcohol.

It is important to note that drink serving sizes are often more than one standard drink. There are no common glass sizes used in Australia.

The label on an alcoholic drink container tells you the number of standard drinks in the container.

TIPS TO REDUCE THE RISK TO YOUR HEALTH WHEN DRINKING

It is possible to drink at a level that is less risky, while still having fun. There are a number of things you can do to make sure you stay within low risk levels and don't get to a stage where you are no longer capable of controlling your drinking.

These include:

- Set limits for yourself and stick to them
- Start with non-alcoholic drinks and alternate with alcoholic drinks
- Drink slowly
- Try drinks with a lower alcohol content
- Eat before or while you are drinking
- If you participate in rounds of drinks try to include some non-alcoholic drinks

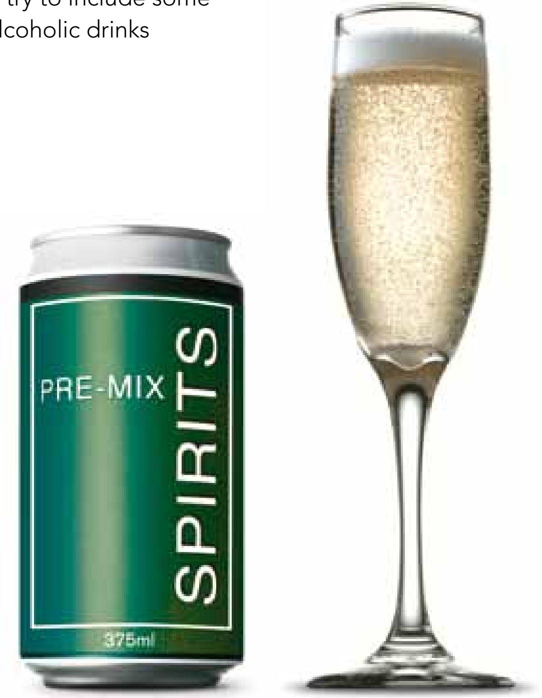

Supplement: Supplementary file 2 — Alcohol Consumption Pamphlet. (PDF 245 kb) [file 13063_2019_3462_MOESM2_ESM.pdf]
