# Supplementary material for: A structured telephone-delivered intervention to reduce problem alcohol use (Ready2Change): study protocol for a parallel group randomised controlled trial
Source: Trials. 2019 Aug 19;20:515. doi: 10.1186/s13063-019-3462-9 (PMC6701125; doi:10.1186/s13063-019-3462-9)
Supplement: Supplementary file 3 — Stress Management Pamphlet. (PDF 113 kb) [file 13063_2019_3462_MOESM3_ESM.pdf]

understanding and managing stress

APS Australian Psychological Society

what is stress?

Stress is often described as a feeling of being overloaded, wound-up tight, tense and worried. We all experience stress at times. It can sometimes help to motivate us to get a task finished, or perform well. But stress can also be harmful if we become over-stressed and it interferes with our ability to get on with our normal life for too long.

what are the signs of stress?

When we face a stressful event, our bodies respond by activating the nervous system and releasing hormones such as adrenalin and cortisol. These hormones cause physical changes in the body which help us to react quickly and effectively to get through the stressful situation. This is sometimes called the 'fight or flight' response. The hormones increase our heart rate, breathing, blood pressure, metabolism and muscle tension. Our pupils dilate and our perspiration rate increases.

While these physical changes help us try to meet the challenges of the stressful situation, they can cause other physical or psychological symptoms if the stress is ongoing and the physical changes don't settle down.

These symptoms can include:

- Headaches, other aches and pains
- Sleep disturbance, insomnia
- Upset stomach, indigestion, diarrhoea
- Anxiety
- Anger, irritability
- Depression
- Fatigue
- Feeling overwhelmed and out of control
- Feeling moody, tearful
- Difficulty concentrating
- Low self-esteem, lack of confidence
- High blood pressure
- Weakened immune system
- Heart disease

types of stress

Acute stress

Sometimes stress can be brief, and specific to the demands and pressures of a particular situation, such as a deadline, a performance or facing up to a difficult challenge or traumatic event. This type of stress often gets called acute stress.

Episodic acute stress

Some people seem to experience acute stress over and over. This is sometimes referred to as episodic acute stress. These kind of repetitive stress episodes may be due to a series of very real stressful challenges, for example, losing a job, then developing health problems, followed by difficulties for a child in the school setting. For some people, episodic acute stress is a combination of real challenges and a tendency to operate like a 'stress machine'. Some people tend to worry endlessly about bad things that could happen, are frequently in a rush and impatient with too many demands on their time, which can contribute to episodic acute stress.

Chronic stress

The third type of stress is called chronic stress. This involves ongoing demands, pressures and worries that seem to go on forever, with little hope of letting up. Chronic stress is very harmful to people's health and happiness. Even though people can sometimes get used to chronic stress, and may feel they do not notice it so much, it continues to wear people down and has a negative effect on their relationships and health.

when to seek professional help

If high levels of stress continue for a long period of time, or are interfering with you enjoying a healthy life, it is advisable to seek professional help. A mental health professional, like a psychologist, can help you identify behaviours and situations that are contributing to high stress, and help you to make changes to the things that are within your control. Seeking help can be one way to manage your stress effectively.

think well be well

Continued over page ▶

tips on how to manage everyday stress

Learning to handle stress in healthy ways is very important. Fortunately, it is easy to learn simple techniques that help. These include recognising and changing the behaviours that contribute to stress, as well as techniques for reducing stress once it has occurred. The following tips from the APS can help you look after your mind and body, and reduce stress and its impact on your health.

Identify warning signs

It is very helpful to be able to identify early warning signs in your body that tell you when you are getting stressed. These vary from person to person, but might include things like tensing your jaw, grinding your teeth, getting headaches, or feeling irritable and short tempered.

Identify triggers

There are often known triggers which raise our stress levels and make it more difficult for us to manage. If you know what the likely triggers are, you can aim to anticipate them and practise calming yourself down beforehand, or even find ways of removing the trigger. Triggers might include late nights, deadlines, seeing particular people, hunger or over-tired children.

Establish routines

Having predictable rhythms and routines in your day, or over a week, can be very calming and reassuring, and can help you to manage your stress.

Routines can include:

- Regular times for exercise and relaxation
- Regular meal times, waking and bedtimes
- Planning ahead to do particular jobs on set days of the week.

Spend time with people who care

Spending time with people you care about, and who care about you, is an important part of managing ongoing stress in your life.

- Spend time with friends and family, especially those you find uplifting rather than people who place demands on you.
- Share your thoughts and feelings with others when opportunities arise. Don't 'bottle up' your feelings.

Look after your health

- Make sure you are eating healthy food and getting regular exercise.
- Take time to do activities you find calming or uplifting, such as listening to music, walking or dancing.
- Avoid using alcohol, tobacco or other drugs to cope.

Notice your 'self-talk'

When we are stressed we sometimes say things in our head, over and over, that just add to our stress. This unhelpful self-talk might include things like: 'I can't cope', or 'I'm too busy', or 'I'm so tired', or 'It's not fair'. While we might think that these are fairly truthful descriptions of what's going on, they are not always helpful to repeat, and can even make you feel worse.

- Notice when you are using unhelpful self-talk, and instead try saying soothing, calming things to yourself to reduce your levels of stress. Try more helpful self-talk like 'I'm coping well given what's on my plate', or 'Calm down', or 'Breathe easy'.
- Keeping things in perspective is also important. When we are stressed, it's easy to see things as worse than they really are. Try self-talk such as 'This is not the end of the world' or 'In the overall scheme of things, this doesn't matter so much'.

Practise relaxation

Make time to practise relaxation. This will help your body and nervous system to settle and readjust. Consider trying some of the following things:

- Learn a formal technique such as progressive muscle relaxation, meditation or yoga.
- Make time to absorb yourself in a relaxing activity such as gardening or listening to music.
- Plan things to do each day that you look forward to and which give you a sense of pleasure, like reading a book.

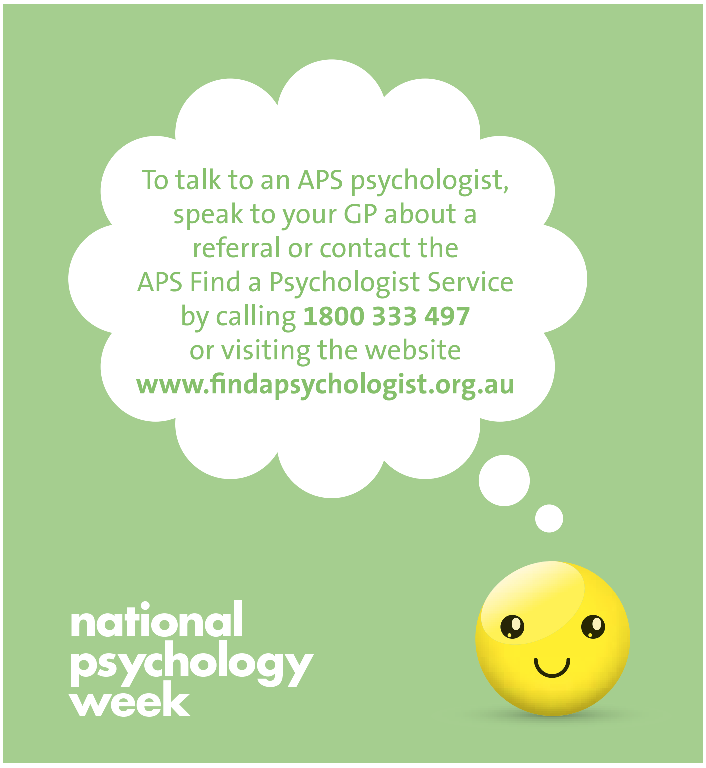

Level 11, 257 Collins Street
Melbourne VIC 3000
PO Box 38
Flinders Lane VIC 8009
T: (03) 8662 3300
F: (03) 9663 6177
www.psychology.org.au

© 2012 The Australian Psychological Society Limited

APS Australian Psychological Society
